# Optimization of the Homogenization Process of Ginseng Superfine Powder to Improve Its Powder Characteristics and Bioavailability

**DOI:** 10.3390/foods13203332

**Published:** 2024-10-20

**Authors:** Mei Sun, Keke Li, Yuanpei Zhang, Jiwen Li, Deqiang Dou, Xiaojie Gong, Zhongyu Li

**Affiliations:** 1 School of Biological Engineering, Dalian Polytechnic University, Dalian 116034, China; sunny201905@yeah.net (M.S.);; 2 College of Life Science, Dalian Minzu University, Dalian 116600, China; 3 College of Pharmacy, Liaoning University of Traditional Chinese Medicine, Dalian 116600, China

**Keywords:** ginseng superfine powder, high-pressure homogenization process, RSM, powder properties, sensory quality

## Abstract

As consumer demands evolve for health supplements, traditional ginseng products are facing challenges in enhancing their powder characteristics and bioavailability. The objective of this study was to prepare a novel ginseng superfine powder using a high-pressure homogenization (HPH) process. Response surface methodology was employed to determine the effects of HPH parameters (pressure, number of passes, and concentration) on particle size and the dissolution of the saponin components of the superfine powders. The Box–Behnken design of experiments was applied to ascertain the optimal HPH parameters for the smallest particle size and the highest dissolution of the saponin components. For the powders obtained at different parameters, the characterization of tap density, bulk density, flowability, water-holding capacity, appearance, and taste were observed. The optimized experimental conditions for the HPH process were as follows: 15,000 psi (pressure), 3 (number of passes), and 1 kg/L (concentration). The optimized values were 55 μm (particle size) and 83 mg/g (dissolution of the saponin components), respectively. The method offered technical support for the application of the HPH process in the preparation of ginseng powders. The objects of this research could be broadened to include a diverse array of botanical materials, addressing contemporary demands for cost-effectiveness and sustainability within the industry.

## 1. Introduction

Ginseng, a perennial herb from the *Panax* genus of the Araliaceae family, has been cherished as a valuable tonic and adaptogen for thousands of years [1]. As the most cultivated, researched and used herb, ginseng is commonly produced as fresh, red, or white ginseng and is sold as powder, capsules, extracts, or in drinks worldwide [2]. Ginseng products are receiving more and more attention, and currently, the global ginseng industry market size has reached 7.61 billion US dollars. With the increase in health awareness and the upgrading of consumer demands, the demand for high-quality ginseng products will continue to grow. It is very important to develop ginseng products that not only deliver health benefits but also offer an improved sensory experience [3]. Innovation in the processing methods of ginseng plays a crucial role in achieving these objectives.

Conventional processing techniques for ginseng, such as slicing, grinding, soaking, compression into tablets, and extraction, can result in several limitations. These include low efficiency in the utilization of beneficial components, a less-than-ideal taste and mouthfeel, and challenges in terms of convenience for the consumer when it comes to ingestion. Superfine grinding is an innovative method in the field of processing that can reduce the particle size of material under 100 μm [4]. The superfine grinding process can endow powders with unique properties that ordinary processing methods cannot produce, such as good solubility, dispersibility, sensory characteristics, and biological activity [5,6], which has been demonstrated in the studies of plants such as goji berries [7], buckwheat [8], and Sanchi [9]. Although scientists have introduced superfine grinding technology into pharmaceutical and food materials [10], the conventional superfine grinding techniques are not well-suited for ginseng due to its hard texture and high fiber content, which make it difficult to achieve the desired particle size through traditional methods. Moreover, the high starch content in ginseng can lead to gelatinization and undesirable physicochemical changes during repeated grinding.

High-pressure homogenization (HPH) presents a viable alternative for the preparation of ginseng superfine powder. As a new type of wet grinding superfine grinding method, HPH has been used in many research areas, such as the food, chemical, pharmaceutical, and biological industries [11]. In the HPH process, the material is suspended in water with high heat capacity, which can effectively prevent starch gelatinization caused by temperature increase. More importantly, HPH can effectively destroy cell wall structures through sudden expansion under high pressure, which maximizes the dissolution of biologically active compounds [12]. Recent studies suggested that the content of flavonoids and polyphenols in bamboo shoots is significantly increased after HPH, accompanied by a decrease in bitterness and an improvement in color [13], and other studies also found similar results [14,15].

Despite these promising findings, the economic feasibility and the scalability of producing ginseng superfine powder through HPH have not yet been realized. The challenges stem from several factors. Firstly, the HPH process is contingent on the interplay of various conditions, which complicates the identification of optimal parameters for large-scale operations. This complexity can lead to inconsistencies in the product quality and efficiency of the process. Additionally, the economic viability is hindered by the high costs associated with the equipment and the energy-intensive nature of the homogenization process. There is also a need for a thorough understanding of the impact of HPH on the physicochemical properties of ginseng, which can influence the product’s marketability and consumer acceptance.

Starting from this gap, the response surface methodology (RSM) was employed to optimize the HPH process for ginseng powder production in the study. RSM is a statistical technique that uses mathematical models to evaluate the relationships between variables and responses, allowing for the determination of optimal conditions. By applying RSM to the HPH process, parameters such as pressure, homogenization cycles, and concentration can be optimized to produce ginseng superfine powder with reduced particle size and enhanced bioactive component utilization. In this article, the relationship between the response values mentioned above and the physicochemical properties and taste characteristics of ginseng superfine powder will also be explored to determine the production process with the best quality parameters. The goal is to highlight the potential of this advanced method in enhancing the modern development of the ginseng industry, ultimately benefiting consumers and producers alike.

## 2. Materials and Methods

### 2.1. Materials

Six years old cultivated ginseng was harvested from Changbai Mountain (Jilin Province, China, Latitude, 41°35′~42°25′ E; Longitude, 127°40′~128°16′ N; Altitude, 2744 m) and then transported to the laboratory by ice-pack within 48 h. All of them were stored in the refrigerator at −20 °C for further application. All other chemical reagents were of analytical grade and purchased from Beijing Chemical Works (Beijing, China).

### 2.2. Design of Experiments

RSM (Stat Ease, MN, USA) was used to design the experiment. Based on the results of early trials, the range of the independent variables, such as pressure (A) (10,000, 15,000, 20,000 psi), number of passes (B) (3, 5, 7), and the substrate concentration (C) (1, 1.5, 2 kg/L) in homogenizer was selected. The Box–Behnken design (BBD) was chosen, and the total number of experiments was 17, including five central point experiments. Table 1 presents the coded and actual values of the independent variables.

### 2.3. Preparation of Ginseng Superfine Powders

**Figure 1:**
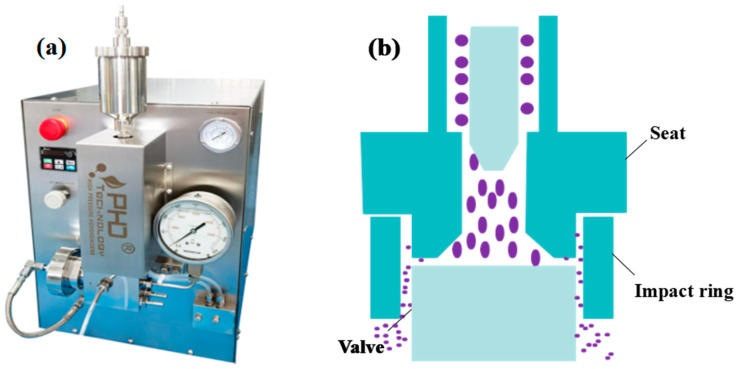
High-pressure homogenizer used in this study (**a**). Operational performance of high-pressure homogenizer (**b**).

The laboratory work was mainly performed a with D-15M high-pressure homogenizer (Figure 1a, PhD Technology LLC, Saint Paul, MN, USA). The machine is composed of a homogenizing valve and pump. The substance entered the homogenizing valve and passed through a homogenization zone of a specific width and then experienced an instantaneous pressure drop and was ejected at an extremely high flow rate, achieving a reduction in particle size through the action of cavitation effect, impact effect, and shearing effect in the homogenization valve [16]. Figure 1b indicates how materials are being disrupted by the effects of the homogenization components.

The fresh ginseng was manually cut into segments measuring 1 cm in length, soaked in a certain amount of pure water, and then ground using a colloid mill as a pretreatment before the homogenization process to reduce the size of fibers to prevent clogging the homogenizing nozzle. The independent variables were adjusted in accordance with the experimental design to obtain different ginseng pulp. The pulp was then placed in a freeze-drying chamber (Sihuan Qihang Technology Co., Ltd., Beijing, China) and freeze-dried under conditions of −25 °C and 50 Pa for 72 h to obtain different ginseng superfine powders. All of them were vacuum-packed in a foil bag and stored at 4 °C for further examination.

### 2.4. Statistical Analysis

The responses obtained were fitted with a quadratic model, followed by conducting an analysis of variance (ANOVA). In addition, the adequacy of the model was determined by the coefficient of determination (R^2^), adjusted R^2^, coefficient of variation (CV), and lack of fit. The *p* value below 0.05 signified that the terms of the model were statistically significant, whereas a *p* value exceeding 0.10 suggested that the model terms were not significant. The predicted response variables (Y) were analyzed using the second-order polynomial regression provided below as Equation (1):

Y = a_0_ + a_1_X_1_ + a_2_X_2_ + a_3_X_3_ + a_12_X_1_X_2_ + a_13_X_1_X_3_ + a_23_X_2_X_3_ + a_11_X_1_^2^ + a_22_X_2_^2^ + a_33_X_3_^2^, (1)

where Y was the predicted response variable, a_0_ was the intercept term, X_i_ was the independent factor coded/actual term, and a_i_ was the model coefficient term.

### 2.5. Optimization

The BBD employed three equal levels—low (coded as −1), medium (coded as 0), and high (coded as +1)—of the independent variables with only 17 sets of experiments. The RSM based on the BBD was utilized to optimize three independent variables. The optimization was performed by setting goals for the independent and dependent variables, which entailed controlling the independent parameters and the responses (particle size and dissolution of the saponin components). The impact of the process variables on the responses was assessed to optimize the process parameters effectively.

### 2.6. Characterization of the Ginseng Powder

#### 2.6.1. Determination of the Diameter

The particle size of ginseng superfine powder was determined using a laser diffraction particle size analyzer (Malvern Instruments Ltd., Worcestershire, UK). Here, the particle size distribution of the ginseng superfine powder was reported as the maximum particle diameter for 97% of the volume of the powder (D_97_). In addition, the specific surface area (Asf) and span value were also noted. Asf referred to the total surface area per unit mass of the material, while span value represented the degree of homogeneity and reliability of particle distribution [7].

#### 2.6.2. Dissolution of the Saponin Components

Ginseng superfine powder (1 g) was dissolved in 60% ethanol (solid-liquid ratio 1:40) for 1 h at 70 °C and subsequently centrifuged at 13,000 rpm for 10 min (TGL-16 high-speed cryogenic centrifuge, Xiangyi Group, Changsha, China). The residue was extracted twice more, and then the three supernatants were combined and evaporated using a rotary evaporator under a vacuum at 60 °C. The evaporated residue was dissolved in 30 mL of distilled water and then extracted with a saturated n-butanol solution three times. A round-bottom flask fitted with a cooling condenser was used to evaporate the n-butanol, and the residue was dissolved in 10 mL of methanol for later use.

Determining dissolution of the saponin components by UV-Vis spectrophotometry based on vanillin-sulfuric acid colorimetry reaction described by Tang et al. with some modifications [17]. The standard curve was plotted using ginsenoside Re as the standard. A total of 500 μL of freshly prepared 8% vanillin-ethanol solution and 5 mL of 72% sulfuric acid to 100 μL of the sample with the solvent dried was added and placed in a 60 °C constant temperature water bath for a reaction of 15 min. The solution was allowed to cool, and the absorbance was measured at 544 nm (SuPerMax 3200 micro-plate reader, Shanpu Co., Ltd., Shanghai, China).

#### 2.6.3. Analyses of Bulk Density, Tap Density, and Flowability

The bulk and tap densities of the ginseng superfine powder were measured according to the method described by Ramachandraiah with some modifications [18]. The sample was filled into a 100 mL graduated cylinder. The weight (mg) of the superfine powder divided by the volume occupied in the cylinder could be used to calculate the bulk density value. The same graduated cylinder was tapped more than 100 times until the volume of the powder stabilized. The weight (mg) of the superfine powder was then divided by the volume to estimate the tap density. The flowability of the sample was evaluated by the Carr index (%) [19]. The Carr index was calculated using Equation (2):

Carr index (%) = (Tap density − Bulk density)/Tap density × 100% (2)

#### 2.6.4. Water-Holding Capacity

The water-holding capacity (WHC) of ginseng superfine powder was determined using a method described by Zhang et al. with some modifications [20]. Approximately 1 g of sample (m_0_) was suspended in 25 g water (m_1_) within a 50 mL centrifuge tube. The powder suspension was then shaken in an electro-thermostatic water bath for 60 min at 60 °C and subsequently centrifuged at 3000 rpm for 10 min. Then, the supernatant (m_2_) was taken out and weighed. WHC value was calculated based on Equation (3):

WHC = (m_1_ − m_2_)/m_0_ × 100% (3)

#### 2.6.5. Morphology

Ginseng superfine powder suspension was uniformly adhered to a metal table with conductive tape until the moisture naturally evaporated. The surface of the sample was sputter-coated with Pt for 15 s using an E-1010 Iron Sputter, and then the morphological characteristics of different samples were observed with a scanning electron microscope (Hitachi Ltd., Tokyo, Japan) operated at an accelerating voltage of 5.0 kV.

#### 2.6.6. Electronic Tongue

The SA402B electronic tongue system (Intelligent Sensor Technology Company, Tokyo, Japan) was used to determine the taste profiles of different ginseng samples. The tongue is composed of an auto-sampler, an array of chemical sensors, and a chemometrics software package. The response types of different sensors for electronic tongues are shown in Table 2. A total of 3 g of ginseng superfine powder was separately dissolved in 150 milliliters of water, and then the mixture was centrifuged at 15,000 rpm for 15 min to collect the supernatant for testing. Test conditions included a sample volume of 120 mL, an analysis time of 90 s, and a time of 336 s. After sensory measurement for each sample solution, a wash cycle was performed to ensure that there was no sample carryover to the next analysis and to ensure good reproducibility. Measurements were performed in triplicate for each sample.

## 3. Results and Discussion

### 3.1. Fitting the Model

RSM was utilized to optimize the process parameters for the production of ginseng superfine powder using a high-pressure homogenizer. The data related to the responses, such as particle size and dissolution of the saponin components, were determined and detailed in the design matrix (Table 3). The response variable (Y) was modeled using a second-order polynomial response surface model. Both regression analysis and ANOVA were employed to estimate the model and assess the statistical relevance of the terms. The ANOVA results, including the statistical parameters, were presented for particle size (Table 4) and the dissolution of the saponin components (Table 5), respectively.

The significance level of each factor was determined by examining the *p* value. The coefficient with a lower *p* value was highly significant. As shown in Table 4, the linear effect of A (Pressure), B (Number of passes), and C (Concentration) and the quadratic effect of A (Pressure) and B (Number of passes) were identified as significant terms within the model. However, the interaction terms showed insignificant effects. As indicated in Table 5, the linear effect of A (Pressure), the quadratic effect of A (Pressure), and the interaction effect of A (Pressure) and C (Concentration) had a significant effect on the dissolution of the saponin components. The term ‘lack of fit’ was a good indication of the failure of the model, and an insignificant ‘lack of fit’ of a model indicated that the model fit the experimental data satisfactorily. The R^2^ value represented the fraction of variability in the response variable that was predictable from the explanatory variables. It was generally recommended that an R^2^ value should be above 0.80 to indicate a well-fitted model, as a value below this threshold suggested that the model might not adequately capture the connections between the variables under study. While a high R^2^ value was often desirable, it was not a definitive indicator of model adequacy. Consequently, assessing the adjusted R^2^ value was crucial, as it offered a more accurate measure of how well the model fitted the data, taking into account the number of predictors in the model [21]. The findings from the ANOVA indicated R^2^ values of 0.9898 and 0.9570 for the particle size and the dissolution of the saponin components, which indicated that the response model could explain 95% of the total variations. Furthermore, the adjusted R^2^ values were found to be 0.9768 and 0.9018 for them. The high R^2^ and adjusted R^2^ values indicated that non-significant terms had not been added to the model. The Adequate precision value represented the signal-to-noise ratio, with a preferred ratio exceeding 4 [22]. Based on the ANOVA results, each response exhibited an Adequate value exceeding 4, which indicated a strong signal and suggested that the model fitting was effective. The coefficient of variation (CV) represented the proportion of the standard error of the estimate relative to the mean of the observed responses, serving as an indicator of the model’s repeatability. The smaller CV values (6.83%, 3.20%) indicated greater precision and dependability of the experimental data. Through the utilization of multiple regression analysis on the experimental data, the relationship between the response variables and the test variables was established using the subsequent second-degree polynomial Equations (4) and (5):

Y*_Particle size_* = 85.40 − 45.63X_1_ − 9.25X_2_ + 29.38X_3_ − 7.00X1X_2_ + 6.75X_1_X_3_ + 2.50X_2_X_3_ + 22.17X_1_^2^ − 7.58X_2_^2^ − 0.825X_3_^2^, (4)

Y*_Dissolution of the saponin components_* = 87.20 + 2.50X_1_ + 0.25X_2_ + 0.5X_3_ − 2.00X_1_X_2_ + 4.00X_1_X_3_ + 0.50X_2_X_3_ − 14.10X_1_^2^ − 2.10X_2_^2^ − 0.10X_3_^2^, (5)

where Y was the response for the particle size and the dissolution of the saponin components. The values obtained from Equations (4) and (5) are presented in Table 3.

The above results showed that the polynomial regression model was in good agreement with the experimental data, suggesting that the model utilized in this study was able to determine the optimal operating conditions for the production of ginseng superfine powder through high-pressure homogenization.

### 3.2. Analysis of the Response Surface

**Figure 2:**
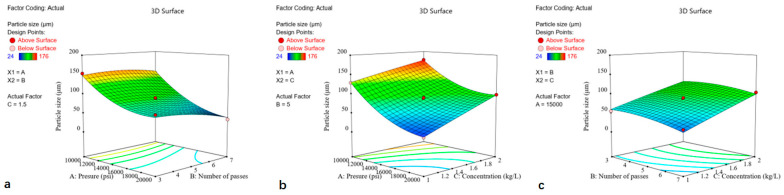
Response surface plot showing the effect of (**a**) pressure and number of passes, (**b**) pressure and concentration, and (**c**) number of passes and concentration on particle size of ginseng superfine powder. The trend showed that increased pressure and decreased concentration resulted in decreased particle size. However, the effect of the number of passes was insignificant. Color pattern in the graph corresponds to the value of particle size where blue (minimum), green (intermediate), and yellow-red (maximum).

**Figure 3:**
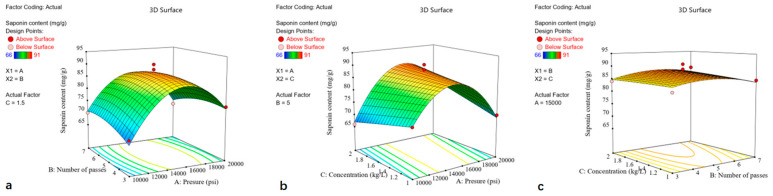
Response surface plot showing the effect of (**a**) pressure and number of passes, (**b**) pressure and concentration, and (**c**) number of passes and concentration on the dissolution of the saponin components of ginseng superfine powder. The trend showed that pressure had a significant impact on the dissolution of total saponin components; however, the effect of the number of passes and concentration was insignificant. Color pattern in the graph corresponds to the value of particle size where blue (minimum), green (intermediate), and yellow-red (maximum).

The effect of the relationship between dependent and independent variables on the particle size and the dissolution of the saponin components was illustrated using a three-dimensional representation of the response surface. The data were generated by holding one variable constant at its intermediate level while varying the other variables across the experimental range under study. The response surface was visualized by particle size (Figure 2) and the dissolution of the saponin components (Figure 3) in relation to the homogenization pressure, number of passes, and concentration.

#### 3.2.1. Particle Size

**Figure 4:**
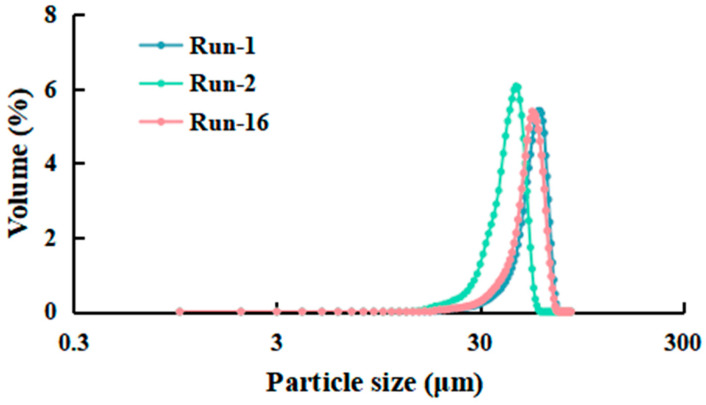
Particle size distributions of ginseng superfine powders randomly selected. The particle size distribution of three randomly selected ginseng superfine powders was as follows: Run-1 (D_97_ 129.83 ± 5.08 μm), Run-2 (D_97_ 24.25 ± 1.08 μm) and Run-16 (D_97_ 98.82 ± 2.08 μm). The chart indicated that the different powders exhibited similar homogeneity.

The particle sizes of the ginseng powders were in the range of 24 μm and 176 μm for experimental run 2 and run 5, respectively. The higher pressure (20,000 psi) and minimum concentration (1 kg/L) resulted in better size reduction, indicating that the pressure and concentration had a significant impact on the reduction in powder particle size. To observe the characteristic particle size distribution of different powders, samples representing low, medium, and high particle size levels (runs 1, 2, and 16) were selected for analysis. As shown in Figure 4, powders with different particle sizes exhibited similar uniformity. The response surface plot showed that the homogenization pressure had a positive linear effect on the particle size of the ginseng superfine powder, whereas the concentration harmed it. However, the effect of the number of passes was much lower, where increasing the homogenization cycle only resulted in a slight decrease in particle size.

Based on the higher pressure, homogenization could effectively reduce particle size by destroying the cell structure through sudden expansion [23,24]. Wang et al. studied the effect of high-pressure homogenization on the reduction of material particle size and indicated that increased pressure could significantly reduce the particle size of cellulose [25]. Davoudpour et al. discovered that an increase in homogenization pressure could lead to a reduction in the diameter of kenaf bast cellulose nanofibers [26]. The high viscosity caused by the increase in concentration might lead to poorer fluidity during the high-pressure homogenization process. The mechanical action of the homogenizer may be diminished when high-viscosity materials pass through the machine’s narrow slits, which can make it challenging to reduce the particle size of these materials during the homogenization process [27].

#### 3.2.2. The Dissolution of the Saponin Components

The dissolution of the saponin components was in the range of 66 mg/g and 91 mg/g. It could be observed that the number of passes and concentration had an insignificant effect on the dissolution of the total saponin components of the ginseng superfine powder through the response surface plot. By comparison, the homogenization pressure had a notable influence on it. The dissolution of the saponin components values increased initially with a pressure of 10,000–15,000 psi and again decreased at pressure from 15,000 to 20,000 psi.

**Figure 5:**
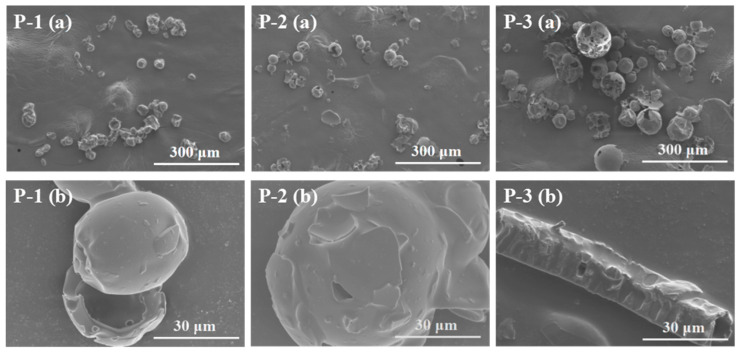
SEM micro-graphs of the powders obtained at low (P-1, Run-2), intermediate (P-2, Run-14), and high (P-3, Run-17) levels of the variables. (**a**,**b**) showed the whole and a section of different powders, respectively.

Ginsenosides are distributed in specific areas, such as cell walls and vacuoles, and are usually covalent bonded with cellular components (polysaccharides, pectin, and cellulose) [28]. The dissolution of the saponin components has a significant correlation with the homogenization pressure owing to the destruction of cell walls and organelle structures (Figure 5), which leads to a marked increase in the release of the active substances bound to them. Wang et al. reported that high-pressure homogenization led to increased release of flavonoids and polysaccharides bond to the cell wall [13]. Jiang et al., in their study, reported that high-pressure homogenization increased the enzymatic hydrolysability in Bamboo shoots [12]. In simple terms, appropriate pressure could increase the dissolution of active substances by disrupting the cell walls and organelles. However, at higher pressures, the increased impact and shear forces might result in an extreme reduction in the powder’s particle size [26]. This smaller particle size resulted in poorer dispersibility, which hindered both wetting and dispersion by the solvent medium and consequently led to a reduction in the dissolution of the active components [29].

### 3.3. Optimization of the Experimental Conditions

To obtain high-quality ginseng superfine powder, the key elements are smaller powder particle size and higher dissolution of the saponin components. Hence, the homogenization pressure, number of passes, and material concentration were optimized to obtain the minimum particle size and the maximum saponin content. The optimum experimental conditions were as follows: the minimum particle size of the powder could be achieved by homogenizing the ginseng pulp five times at a concentration of 1 kg/L under a pressure of 20,000 psi, the maximum dissolution of the saponin components content could be obtained by homogenizing the ginseng pulp five times at a concentration of 1.5 kg/L under a pressure of 15,000 psi. It was evident that the optimized experimental conditions varied for the particle size and the dissolution of the saponin content. Consequently, it was deemed valuable to determine a single operational condition to gain ginseng superfine powder with the best physical and chemical properties. Performance parameters of the powders obtained at low (P-1, run 2), intermediate (P-2, run 14), and high (P-3, run 17) levels of the variables were analyzed.

#### 3.3.1. Bulk Density, Tap Density, and Flowability

Bulk density, tap density, and flowability are important parameters for evaluating the powder quality in the food industry [30]. Powders with higher bulk and tap density could more effectively utilize packaging space, whereas those with lower densities were easier to dissolve and disperse [31]. In industrial production, it is necessary to determine the moderate densities of the powder based on its intended use to achieve a balance between the storage costs and uniformity of the powder. Generally speaking, the smaller the particle size of the powder, the smaller the pore spaces between the particles, which in turn results in a higher density [32]. As shown in Table 6, the bulk density of P-1 was slightly higher than that of the other two powders, and the tap density followed the same trend. These results were in agreement with the above conclusion. However, there was no significant difference between the bulk and tap densities of these three superfine powders, which indicated that the changes in the homogeneity parameter had little effect on them.

The Carr index was also used to evaluate the fluidity of powdered products. The Carr index demonstrated a slight increase with corresponding decreases in powder particle size [33]. Carr index values ranging from 0–15% were considered as good flowability, 15–25% fell in the range for fair flowability, 25–30% were considered in the poor range, and over 30% were considered as very poor flowability [34]. As depicted in Table 6, a fair degree of flowability was observed for P-1 (Carr index: 23.23%), P-2 (Carr index: 20.64%), and P-3 (Carr index: 19.79%).

#### 3.3.2. Water-Holding Capacity (WHC)

**Figure 6:**
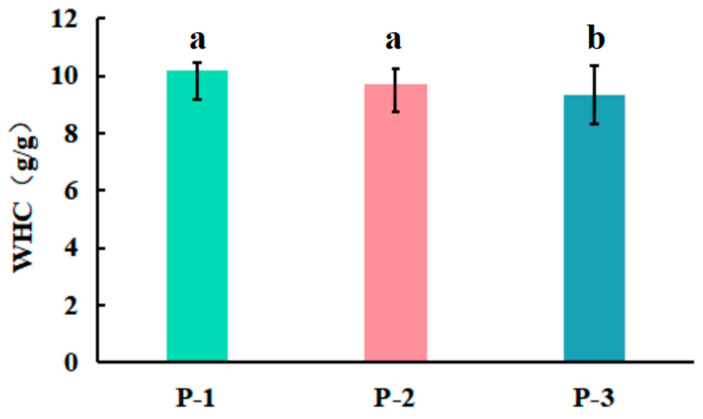
Effect of WHC value of the powders obtained at low (P-1, Run-2), intermediate (P-2, Run-14), and high (P-3, Run-17) levels of the variables (Data represent mean values ± standard deviation. Different lowercase letters show significant differences (*p* < 0.05, n = 3)).

The WHC value reflected the binding capacity of powder and water, which was related to the ability of powder to retain moisture when subjected to centrifugal force or compression [35]. Powders with a higher WHC value usually had a smoother and finer texture in the food and beverage industry. Additionally, they exhibited enhanced solubility and dispersibility by absorbing moisture when mixed with liquids [36]. As shown in Figure 6, the WHC of P-3 was lower than those of the other two samples (*p* < 0.05); P-2 and P-1 showed no difference in the value (*p* > 0.05). In brief, the P-1 and P-2 samples exhibited a better water-holding capacity.

#### 3.3.3. Morphology

The structure change of ginseng superfine powders obtained at different levels of the variables in morphology were analyzed using SEM. As shown in Figure 5a,b showed the whole and a section of different powders, respectively. It could be observed in Figure 5a that the particle size of P-1, P-2, and P-3 gradually increased, which was consistent with the analysis results of the particle size analyzer. Specifically, aggregation was evident in P-1 powders, which were formed by smaller particles or fragments adhered to larger ones. The dissolution of saponin components would reduced because of the accumulation of powders [9], which was consistent with the results of the study above. In comparison, the tissue structure of P-2 was essentially destroyed, causing fibers to exhibit irregular particles within the field of view. However, plant tissues in P-3 exhibited a relatively intact appearance. A further zoomed image (Figure 5b) showed that there was no completed cell structure in the P-1 and P-2 samples. At the same time, the ginseng particles displayed a solid surface with cracks, which was the reason for the differences in the dissolution of active compounds [37]. However, the intact cork cells and resin channel fragments could still be seen in the P-3 visual field.

#### 3.3.4. Analysis of the Taste Features by E-Tongue

Electronic tongue, for objective evaluation such as the discrimination and quantification of tastes, was known as a sensing technology that contributes to quality management [38]. The electronic tongue was recently been used to evaluate the tastes of various traditional Chinese medicine, such as *Polygonum multiflorum* [39], berberine [40], Pericarpium Citri Reticulate [41], and *Schisandra sphenanthera* [42].

**Figure 7:**
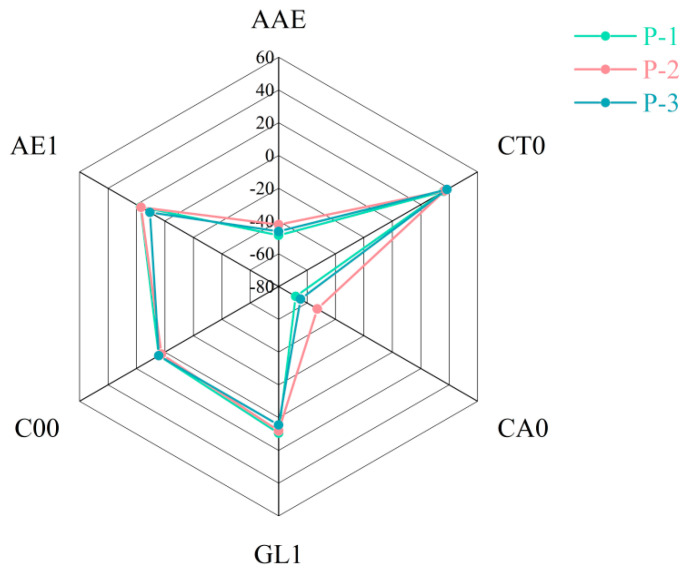
Electronic tongue radar analysis plots of the powders obtained at low (P-1, Run-2), intermediate (P-2, Run-14), and high (P-3, Run-17) levels of the variables.

In order to investigate whether different homogenization conditions would affect the taste features of ginseng superfine powder, the electronic tongue was used to evaluate the indices of tastes. There were six different sensors in the SA402B electronic tongue system. Radar chromatograms were drawn based on the response values of three groups of ginseng superfine powders on these six sensors, where different colors represented different ginseng superfine powders. As shown in Figure 7, the saltiness and astringency sensors had the highest response value to superfine powder samples in each group, while the sour taste had the lowest response value among all the sensors. There were no significant differences in taste among the three groups, except for slight variations in sourness and astringency. In short, the overall taste of the three groups of samples was similar, and they could be more obviously categorized as belonging to the same category.

## 4. Conclusions

The work was purposed to prepare a ginseng superfine powder with superior powder characteristics and bioavailability by an HPH process, optimized using RSM. The effects of high pressure, number of passes, and concentration on the particle size and total saponin solubility of ginseng superfine powder were investigated through the BBD experiment. A comparison was conducted on the powder characteristics of three representative superfine powders (labeled as P-1, P-2, and P-3), including tap density, bulk density, fluidity, WHC, appearance, and taste. The results of the experimental and statistical analysis showed that powders P-1 and P-2 exhibited superior performance in all the powder characteristics evaluated, which were crucial for the functionality and consumer acceptance of powder products. Production costs are closely related to the number of cycles. The production conditions of P-2 (Pressure: 15,000 Pai, Number of passes: 3, Concentration: 1 kg/L) were determined to be the optimal process parameters after considering production-related factors. At the optimized conditions, the responses of particle size (55 μm) and total saponin dissolution (83 mg/g) were recorded, which possessed excellent powder and taste characteristics.

The method presented in this paper has a certain degree of universality, which has great significance for guiding the production of superfine powders from plants rich in high fiber and starch. The research can also be expanded to other types of traditional Chinese medicinal materials to achieve a broader range of applications and meet the requirements for cost-effectiveness and sustainability.

Since the study only tested the dissolution of the saponin components, based on the diversity of ginseng active components, more kinds of active groups should be evaluated to systematically explore the impact of ultra-fine grinding on the samples. Additionally, based on the variety of biological activities of ginseng, subsequent bioactive functional experiments can be carried out on powder with different particle sizes, aiming to expand the application potential of ginseng superfine powders in the field of functional food.

## Figures and Tables

**Table 1:** BBD matrix for the experimental run with actual and coded values (in parenthesis).

Independent Variables	Coded (In Parenthesis) and Actual Levels		
	(−1)	(0)	(1)
Pressure (A), psi	10,000	15,000	20,000
Number of passes (B), n	3	5	7
Concentration (C), kg/L	1	1.5	2

**Table 2:** Response types of different chemical sensors for electronic tongues.

Number	Sensor	Response Types
1	AAE	umami
2	CT0	saltiness
3	CA0	sourness
4	C00	bitterness
5	AE1	astringency
6	GL1	sweetness

**Table 3:** Three factor three level BBD matrix and responses ^a^.

Std	Run	Factor A Pressure (psi)	Factor B Number of Passes (n)	Factor C Concentration (kg/L)	Particle Size (μm)	Dissolution of the Saponin Components (mg/g)
1	7	10,000	3	1.5	153	68
2	11	20,000	3	1.5	76	76
3	4	10,000	7	1.5	138	70
4	13	20,000	7	1.5	33	70
5	1	10,000	5	1	129	74
6	2	20,000	5	1	24 *	72
7	5	10,000	5	2	176 **	66 *
8	16	20,000	5	2	98	80
9	14	15,000	3	1	55	83
10	12	15,000	7	1	42	85
11	15	15,000	3	2	107	84
12	6	15,000	7	2	104	88
13	8	15,000	5	1.5	87	89
14	9	15,000	5	1.5	86	86
15	3	15,000	5	1.5	90	85
16	17	15,000	5	1.5	83	91 **
17	10	15,000	5	1.5	81	85

^a^,* corresponds to minimum value and ** corresponds to maximum value.

**Table 4:** Analysis of variance with linear, quadratic, and interaction effects of the independent variables on particle size ^a,b^.

Source	Sum of Squares	df	Mean Square	F-Value	*p*-Value	
**Model**	26,887.81	9	2987.53	75.78	<0.0001	significant
A-Pressure	16,653.13	1	16,653.13	422.44	<0.0001	
B-Number of passes	684.5	1	684.5	17.36	0.0042	
C-Concentration	6903.12	1	6903.12	175.11	<0.0001	
AB	196	1	196	4.97	0.061	
AC	182.25	1	182.25	4.62	0.0686	
BC	25	1	25	0.6342	0.452	
A^2^	2070.44	1	2070.44	52.52	0.0002	
B^2^	241.6	1	241.6	6.13	0.0425	
C^2^	2.87	1	2.87	0.0727	0.7952	
**Residual**	275.95	7	39.42			
Lack of Fit	226.75	3	75.58	6.14	0.0559	not significant
Pure Error	49.2	4	12.3			
**Cor Total**	27,163.76	16				

^a^ R^2^ = 0.9898, Adjusted R^2^ = 0.9768, Adeq Precision = 31.1494, CV (%) = 6.83. ^b^ *p* < 0.05 indicates that the model terms are significant.

**Table 5:** Analysis of variance with linear, quadratic, and interaction effects of the independent variables on the dissolution of the saponin components ^a,b^.

Source	Sum of Squares	df	Mean Square	F-Value	*p*-Value	
**Model**	1008.94	9	112.1	17.32	0.0005	significant
A-Pressure	50	1	50	7.73	0.0273	
B-Number of passes	0.5	1	0.5	0.0773	0.7891	
C-Concentration	2	1	2	0.3091	0.5956	
AB	16	1	16	2.47	0.1599	
AC	64	1	64	9.89	0.0163	
BC	1	1	1	0.1545	0.7059	
A^2^	837.09	1	837.09	129.35	<0.0001	
B^2^	18.57	1	18.57	2.87	0.1341	
C^2^	0.0421	1	0.0421	0.0065	0.938	
**Residual**	45.3	7	6.47			
Lack of Fit	16.5	3	5.5	0.7639	0.5705	not significant
Pure Error	28.8	4	7.2			
**Cor Total**	1054.24	16				

^a^ R^2^ = 0.9570, Adjusted R^2^ = 0.9018, Adeq Precision = 10.7376, CV (%) = 3.20. ^b^ *p* < 0.05 indicates that the model terms are significant.

**Table 6:** Effect of particle size on the bulk density, tap density, and flowability ^a,b^.

Sample	Bulk Density (mg/mL)	Tap Density (mg/mL)	Carr Index (%)
P-1	471.49 ± 20.81	615.76 ± 20.71 ^a^	23.23
P-2	453.53 ± 11.88	572.57 ± 20.96 ^a^	20.64
P-3	452.79 ± 19.33	552.88 ± 11.25 ^b^	19.79

^a^ Data represent mean values ± standard deviation. (n = 3) ^b^ Different lowercase letters show significant differences. (*p* < 0.05).

## Data Availability

The original contributions presented in the study are included in the article, further inquiries can be directed to the corresponding author.
